# Microglia Activated by Excess Cortisol Induce HMGB1 Acetylation and Neuroinflammation in the Hippocampal DG Region of Mice Following Cold Exposure

**DOI:** 10.3390/biom9090426

**Published:** 2019-08-30

**Authors:** Bin Xu, Li-Min Lang, Shi-Ze Li, Jing-Ru Guo, Jian-Fa Wang, Huan-Min Yang, Shuai Lian

**Affiliations:** College of Animal Science and Veterinary Medicine, Heilongjiang Bayi Agricultural University, Daqing 163319, China

**Keywords:** cold stress, excess cortisol, HMGB1 acetylation, hippocampus, neuroinflammation

## Abstract

Cold stress can induce neuroinflammation in the hippocampal dentate gyrus (DG), but the mechanism underlying neuronal apoptosis induced by cold stress is not well-understood. To address this issue, male and female C57BL/6 mice were exposed to a temperature of 4 °C for 3 h per day for 1 week, and glial cell activation, neuronal apoptosis, and neuroinflammation were evaluated by western blotting, immunofluorescence, terminal deoxynucleotidyl transferase 2’-deoxyuridine 5’-triphosphate (dUTP) nick end labeling, Nissl staining, and immunohistochemistry. Additionally, BV2 cells were treated with different concentrations of cortisol (CORT) for 3 h to mimic stress and molecular changes were assessed by western blotting, immunofluorescence, and co-immunoprecipitation. We found that excess CORT activated glial cells and increased neuroinflammation in the DG of mice exposed to cold temperatures, which was associated with increased acetylation and nuclear factor-κB signaling. These effects were mediated by the acetylation of lysine 9 of histone 3 and lysine 310 of p65, which resulted in increased mitogen-activated protein kinase phosphorylation, nuclear translocation of p65, microglia activation, and acetylation of high-mobility group box 1. Neuroinflammation was more severe in male compared to female mice. These findings provide new insight into the mechanisms of the cold stress response, which can inform the development of new strategies to combat the effects of hypothermia.

## 1. Introduction

The stress response is a common nonspecific response to endogenous and exogenous environmental challenges, [1,2] such as low temperature, which can affect neuroendocrine and metabolic processes. The hypothalamic-pituitary-adrenal (HPA) axis is the primary regulator of the stress response, which leads to glucocorticoid secretion and influences immunity and metabolism, in addition to other central and peripheral functions [2]. Hyperactivation of the HPA axis caused by various stressors can lead to abnormal glucocorticoid secretion, resulting in irreversible damage to the hippocampus [3], a brain region that is important for memory and learning [4]. The hippocampus is an important brain structure that is highly sensitive to stress, which can alter the function of the CA1, CA3, and DG regions [5], especially adult granule cells in the DG region that are involved in learning [6]. Neuroinflammation is a common response to stress that is mediated by glucocorticoid-activated microglia and has been implicated in the etiology of neurodegenerative diseases [7]. Cold exposure is an unavoidable stressor in extreme environments that can induce cold stress, which can lead to a loss of cortical bone [8], alter the gut microbiome profile [9], and influence insulin sensitivity [10]. Moreover, cold stress has been linked to Alzheimer’s disease, although the underlying mechanism is unclear [11]. In our previous study, we showed that neuroinflammation in the CA1 and CA3 regions of the hippocampus following cold exposure is differentially regulated in male vs. female mice [12].

High-mobility group box (HMGB)1 is a major regulator of innate immunity and can induce aseptic inflammation as a damage-associated molecular pattern (DAMP) in response to stress [13]. HMGB1 was originally identified as a non-histone DNA-binding protein localized in the nucleus that maintains the nucleosome structure, regulates gene transcription, and modulates transcription, including that of the glucocorticoid receptor [14]. It was recently shown that HMGB1 promotes neuroinflammation via the pattern recognition receptors Toll-like receptor (TLR)2 and TLR4 and the receptor for advanced glycation end products [15]. HMGB1 is passively released from damaged cells or actively secreted by immunocompetent cells such as microglia, monocytes, and macrophages [16]. However, in the former instance, HMGB1 in apoptotic cells is not recognized by the innate immune system since it is sequestered in the cytosol [17]. The second mechanism involves the hyperacetylation of lysine HMGB1 and its transport from the nucleus to the cytoplasm, which culminates in its release from the cell [18]. Microglia activation reflects the disturbance of homeostasis, which could lead to the release of various factors, including HMGB1. Some studies have reported that HMGB1 expression is increased in mouse tissues following stress through mechanisms that remain unclear.

In our previous study, we demonstrated that the corticosterone (CORT) level and HMGB1 acetylation are increased and microglia are activated in the DG region of the hippocampus of mice following cold exposure [19]. However, the molecular basis of microglia activation and increased HMGB1 acetylation in the mouse hippocampus following cold exposure is not well-understood. We speculated that abnormalities in glucocorticoid secretion due to stress-induced activation of the HPA axis and microglia lead to HMGB1 acetylation and secretion and consequently, neuroinflammation. This was investigated in the present study in mice exposed to cold temperatures and in BV2 cells treated with different concentrations of CORT to simulate the stress response.

## 2. Materials and Methods

### 2.1. Animals and Experimental Design

A total of 50 male C57BL/6 mice (5 weeks old, 22–24 g) were purchased from Charles River (Beijing, China) and randomly divided by sex into the following four groups (*n* = 12 per group): room temperature male (RTM), room temperature female (RTF), cold exposure male (CEM), and cold exposure female (CEF). The different groups were maintained under the same conditions in each experiment. The conditions of cold exposure have been previously described [20]. Each group was pre-treated in a climatic chamber at an ambient temperature of 24 °C ± 2 °C and 40% relative humidity under a 12/12-h light/dark cycle (lights on from 08:00–20:00) with free access to food and water for 1 week. CEM and CEF groups were transferred to a climatic chamber at 4 °C for 3 h per day, and then returned to the original environment at 24 °C ± 2 °C and 40% relative humidity between the hours of 08:00–20:00. Mice in the room temperature (RT) group were maintained at 24 °C ± 2 °C and 40% relative humidity. The cold exposure cycle lasted for 7 days. All experimental procedures were approved the Management Committee of the Experimental Animal Center of Heilongjiang Bayi Agricultural University (5 February 2019).

### 2.2. Serum and Hippocampus Corticosterone Assay

To evaluate the CORT status of the mouse serum and hippocampus after cold exposure, male and female mice (*n* = 5 per group) were immediately anesthetized with pentobarbital and sacrificed by decapitation, after which blood samples and the hippocampus samples were removed from the cold exposure (CE) and room temperature (RT) groups. The hippocampus tissue homogenate was centrifuged at 1000× *g* for 20 min, and the supernatants were collected. Serum CORT levels and the hippocampus CORT levels were measured using a commercial ELISA kit following the manufacturer’s instructions (USCN Life, Wuhan, China).

### 2.3. Brain Tissue Collection

After the last cold exposure cycle, mice were immediately anesthetized with pentobarbital and transcardially perfused with normal saline and 4% paraformaldehyde. The brain was immediately removed and fixed in 4% paraformaldehyde for 48 h, immersed in a 30% sucrose solution for 24 h, flash frozen in liquid nitrogen, and cut into 30-μm-thick serial coronal sections (*n* = 10 per brain) on a freezing microtome (Leica, Wetzlar, Germany; CM1850) that were stored at −80 °C until use. For western blot analysis (*n* = 6 per group), the hippocampus was isolated, washed in ice-cold phosphate-buffered saline (PBS), and stored at −80 °C until use.

### 2.4. Immunofluorescence Analysis

Brain sections were rinsed with PBS, treated with 0.3% H_2_O_2_ for 15 min, and rinsed in PBS. The sections were then blocked with 1% goat serum albumin (Solarbio, Beijing, China; SL039) for 10 min at room temperature and then incubated overnight at 4 °C with an antibody against cluster of differentiation (CD)11b (Abcam, Cambridge, MA, USA; ab1211, 1:100) or Allograft inflammatory factor 1 (IBA-1) (Proteintech, Rosemont, IL, USA, #10904-1-AP, 1:100). After rinsing with PBS, a secondary antibody CL488-conjugated Affinipure donkey anti-mouse Immunoglobulin G (IgG) (H+L); (#SA00006-5, 1:200, Proteintech) or CoraLite594-conjugated Donkey Anti-Mouse IgG (H+L); (#SA00013-7, 1:200, Proteintech) was applied for 1 h at room temperature. Nuclei were counterstained with 4’,6-diamidino-2-phenylindole. Sections were observed under a laser scanning confocal microscope (Leica; TCS SP2) and CD11b- and IBA-1-positive cells were counted in each group at the same magnification.

### 2.5. Immunohistochemistry

Brain sections were rinsed with PBS, treated with 0.3% H_2_O_2_ for 15 min, and rinsed three times for 5 min each in PBS. The sections were blocked with 1% goat serum albumin for 10 min at room temperature and then incubated overnight at 4 °C with an antibody against glial fibrillary acidic protein (GFAP) (16825-1-AP, 1:1000) or microtubule-associated protein (MAP)2 (17490-1-AP, 1:100) (Proteintech). The sections were rinsed and incubated with biotin-conjugated Affinipure goat anti-rabbit IgG (H+L) (#SA00004-2, 1:500; Proteintech) for 1 h at room temperature, followed by diaminobenzidine (Solarbio; DA1010). The sections were dehydrated through an alcohol gradient, cleared in xylene, and mounted for observation with a light microscope (Leica; DMI 5000M). GFAP-positive cells were counted in each group under the same magnification and the optical density was analyzed with ImageJ software (National Institutes of Health, Bethesda, MD, USA).

### 2.6. Nissl Staining

Nissl staining was performed with Nissl Staining Solution (Beyotime Institute of Biotechnology, Shanghai, China; C0117) according to the manufacturer’s instructions. Nissl-positive cells were viewed under a microscope.

### 2.7. Terminal Deoxynucleotidyl Transferase dUTP Nick End Labeling (TUNEL) Assay

Brain sections were rinsed with PBS, treated with 0.3% H_2_O_2_ for 15 min, and rinsed three times for 5 min each in PBS, followed by incubation in TUNEL Stain Solution (Beyotime Institute of Biotechnology; C1086), according to the manufacturer’s instructions. The sections were imaged with a laser scanning confocal microscope and TUNEL-positive cells were counted in each group under the same magnification.

### 2.8. Cell Culture and Reagents

Mouse BV2 cells were a gift from Professor Liu (College of Veterinary Medicine, Jilin University, Jilin, China) and were maintained in Dulbecco’s Modified Eagle’s Medium supplemented with 10% fetal bovine serum (Gibco, Grand Island, NY, USA) at 37 °C and 5% CO_2_ in a humidified incubator. Cells were grown in a monolayer and routinely passaged daily.

### 2.9. CORT Treatment

BV2 cells were incubated with different concentrations of CORT (Sigma-Aldrich, St. Louis, MO, USA) dissolved in dimethyl sulfoxide (Solarbio) for 3 h to obtain a stock solution, which was diluted to obtain working solutions that were used to establish an in vitro stress model.

### 2.10. Cell Counting Kit (CCK)-8 Assay

The effect of CORT on cell viability was determined with the CCK-8 assay. BV2 cells were treated with CORT (0–500 μM) for 3 h. A 10-μL volume of CCK-8 (Beyotime Institute of Biotechnology) was added to each well and after 1 h, cell viability was evaluated by measuring the absorbance at 450 nm on a microplate reader.

### 2.11. Quantitative Real-Time (qRT-)PCR Analysis

Total RNA was isolated from BV2 cells with TRIzol reagent (Invitrogen, Carlsbad, CA, USA), with or without CORT treatment, and the mRNA level of interleukin (IL)-1β was evaluated by qRT-PCR using the primer sequences shown in Table 1.

### 2.12. Hippocampus Tissue and Cell Protein Extraction

Total protein was extracted from the hippocampus with 150 μL radioimmunoprecipitation (RIPA) buffer (Beyotime Institute of Biotechnology) containing 15 mM phenylmethylsulfonyl fluoride (PMSF) (Beyotime Institute of Biotechnology). Total cell protein was extracted from cells treated with 200 μM CORT using 100 μL RIPA buffer (Beyotime Institute of Biotechnology) containing 10 mM PMSF. The samples were stored at −80 °C until western blot analysis. Protein concentration was determined with the Enhanced BCA Protein Assay Kit (Beyotime Institute of Biotechnology), according to the manufacturer’s instructions.

### 2.13. Cytoplasmic and Nuclear Protein Extraction

Nuclear proteins were extracted from cell samples treated with 200 μM CORT using the Nuclear and Cytoplasmic Protein Extraction Kit (Beyotime Institute of Biotechnology) and the protein concentration was determined as described above.

### 2.14. Western Blot Analysis

Approximately 30 μg of total protein was separated by sodium dodecyl sulfate–polyacrylamide gel electrophoresis and transferred to polyvinylidene difluoride membranes (0.22 and 0.45 μm; Millipore, Darmstadt, Germany) that were blocked for 1 h at room temperature in 5% nonfat milk in Tris-buffered saline containing 0.1% Tween 20 (TBST), which were then incubated overnight at 4 °C with antibodies against the following proteins: HMGB1 (#21865-1-AP, 1:3000), GFAP (#16825-1-AP, 1:2000), ionized calcium-binding adapter molecule (IBA)-1 (#10904-1-AP, 1:500), p65 (#10745-1-AP, 1:1000), silent mating type information regulation 2 homolog (SIRT)1 (#66292-1-Ig, 1:3000), brain-derived neurotrophic factor (BDNF); #16806-1-AP, 1:1000), IL-1β (#18985-1-AP, 1:1000), inducible nitric oxide synthase (iNOS) (#12987-1-AP, 1:1000), lamin B1 (#17168-1-AP, 1:3000), histone H3 (#60008-1-lg, 1:15,000), and β-actin (#14395-1-AP, 1:15,000) (all from Proteintech); and extracellular signal-regulated kinase (ERK) (#4695S, 1:1000), phospho-ERK (Thr202/Tyr204) (#9101, 1:1000), c-Jun N-terminal kinase (JNK) (#9252, 1:1000), phospho-JNK (Thr183/Tyr185) (#4668, 1:1000), p38 (#8690, 1:1000), phospho-p38 (Thr180/Tyr182) (#4511, 1:1000), acetyl-p65 (Lys310) (#12629S, 1:1000), and acetyl-histone H3 (Lys9) (#9649, 1:1000) (all from Cell Signaling Technology, Danvers, MA, USA). The membrane was rinsed five times with TBST for 10 min each time and then incubated for 1 h at room temperature with the following horseradish peroxidase (HRP)-conjugated secondary antibodies: Affinipure goat anti-mouse IgG (H+L) (SA00001-1, 1:8000) and goat anti-rabbit IgG (H+L) (SA00001-1, 1:8000) (both from Proteintech). After rinsing, the membrane was treated with Chemiluminescent HRP Substrate (Millipore) and proteins bands were detected with a chemiluminescence detector (Bio-Rad, Hercules, CA, USA). Protein expression levels were measured using Image Lab software (Bio-Rad).

### 2.15. Immunocytochemistry

BV2 cells were seeded on slides and incubated for 24 h at 37 °C, followed by incubation with 200 μM CORT for 24 h; they were then fixed with 4% paraformaldehyde and seeded on poly-l-lysine-coated coverslips. The cells were permeabilized with 0.3% Triton X-100, blocked with 3% bovine serum albumin, and incubated overnight at 4 °C with antibodies against CD11b (abcame; #ab1211, 1:100), IBA-1 (Proteintech; #10904-1-AP, 1:100), and HMGB1 (Proteintech; 10829-1-AP, 1:100), followed by CL488-conjugated Affinipure donkey anti-mouse IgG (H+L) (Proteintech; SA00006-5, 1:200). Nuclei were stained with 4’,6-diamidino-2-phenylindole and the slides were viewed with a laser scanning confocal microscope (Leica; TCS SP2). The number of CD11b, IBA-1-, and HMGB1-positive cells was counted.

### 2.16. Co-Immunoprecipitation (Co-IP)

Co-IP of HMGB1, acetyl-lysine, acetyl-histone H3 (Lys9), and p65 was performed using a Dynabead Protein An Immunoprecipitation Kit (Thermo Fisher Scientific, Waltham, MA, USA; 10006D), according to the recommended protocol. In BV2 cells, the beads were pre-cleared with anti-acetyl-histone H3 (Lys9) (#9649, 1:25; Cell Signaling Technology) and anti-acetylated-lysine (#9441, 1:50; Cell Signaling Technology) antibodies for 10 min, and proteins in hippocampal tissue or cell lysate were extracted using Nonidet P-40 lysis buffer (Beyotime Institute of Biotechnology); 60 µg of sample was incubated with the beads for 15 min prior to elution, and HMGB1 and p65 levels in the eluent were assessed by western blotting, as described above.

### 2.17. Statistical Analysis

Statistical analyses were performed using Prism v.7.0 software (GraphPad Inc., La Jolla, CA, USA). Values are expressed as the mean ± SD. Statistical comparisons were performed across different treatment groups (room temperature and cold exposure) and between sexes (male and female) by two-way analysis of variance and in vitro data were analyzed by one-way analysis of variance. *p* < 0.05 was considered statistically significant.

## 3. Results

### 3.1. CORT Levels in the Mouse Serum and Hippocampus Following Cold Exposure

**Figure 1:**
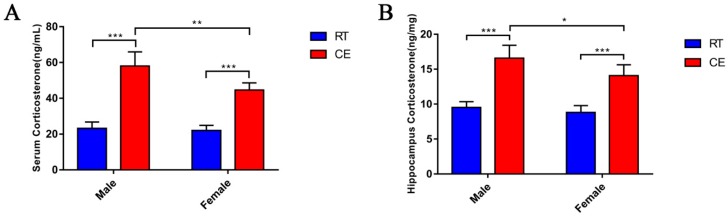
Serum and hippocampus tissue cortisol (CORT) levels of male and female mice at room temperature male (RTM), cold exposure male (CEM), room temperature female (RTF), and cold exposure female (CEF) mice. Data analyses in different treatment (**A**) and sex groups (**B**,**C**) by two-way ANOVA. Results are expressed as the mean ± SD (*n* = 10) in ten independent experiments. N.S.: not significant; * *p* < 0.05, ** *p* < 0.01, *** *p* < 0.001.

In order to evaluate the effect of CORT on the mice hippocampus following cold exposure, the levels of CORT in the hippocampus and serum of mice following cold exposure were measured. The CORT levels were significantly increased in the hippocampus and serum of the CE groups of male and female mice when compared with the RT groups. Moreover, the CORT levels were significantly increased in the CEM group when compared to the CEF group (Figure 1A,B).

### 3.2. Cold Exposure Increases the Number of Glial Cells in the Hippocampus

**Figure 2:**
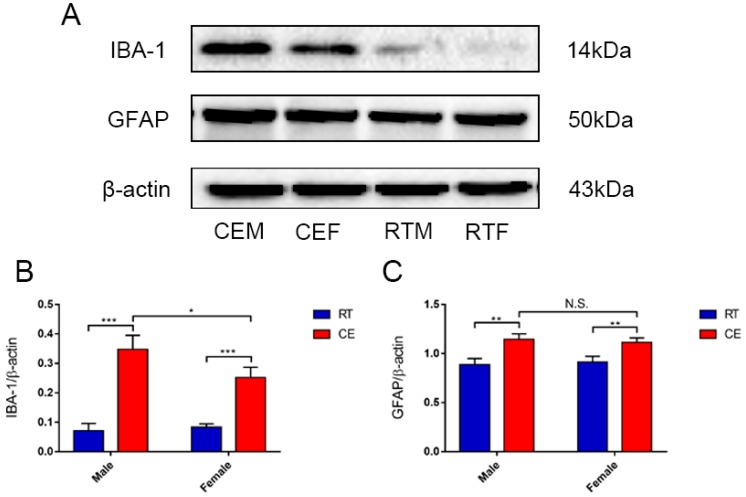
Expression of glial cell makers in hippocampal tissue lysates of cold-stressed mice, as determined by western blotting and densitometric analysis. (**A**) IBA-1, glial fibrillary acidic protein (GFAP), and β-actin levels in each group. (**B**,**C**) Quantification of expression levels based on expression ratios of IBA-1/β-actin (**B**) and GFAP/β-actin (**C**). Differences between room temperature male (RTM) and cold exposure male (CEM), room temperature female (RTF) and cold exposure female (CEF), and CEM and CEF groups were evaluated by two-way analysis of variance. Results are expressed as the mean ± SD (*n* = 6) of four independent experiments. N.S.: not significant; * *p* < 0.05, ** *p* < 0.01, *** *p* < 0.001.

**Figure 3:**
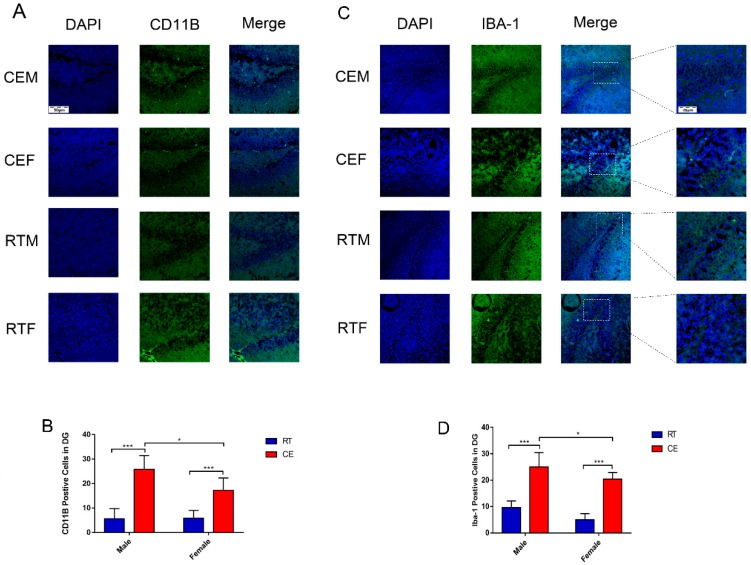
Immunofluorescence detection of the activated microglia marker CD11b and IBA-1 in the dentate gyrus (DG) of cold-stressed mice. (**A**) CD11b-positive cells (green) were counterstained with 4’,6-diamidino-2-phenylindole (blue). Scale bar = 50 μm. (**B**) Quantification of CD11b-positive cells in the DG. (**C**) IBA-positive cells (green) were counterstained with 4’,6-diamidino-2-phenylindole (blue). Scale bar = 50 or 25 μm. (**D**) Quantification of IBA-1-positive cells in the DG. Differences between room temperature male (RTM) and cold exposure male (CEM), room temperature female (RTF) and cold exposure female (CEF), and CEM and CEF groups were evaluated by two-way analysis of variance. Results are expressed as the mean ± SD (*n* = 6) of four independent experiments. * *p* < 0.05, *** *p* < 0.001.

**Figure 4:**
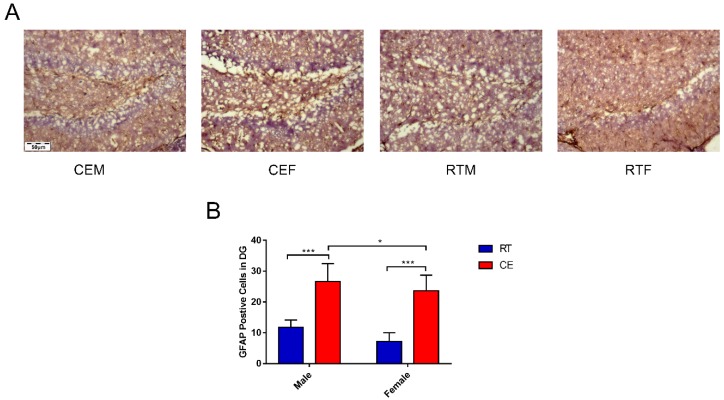
Immunohistochemical detection of the astrocyte marker glial fibrillary acidic protein (GFAP) in the dentate gyrus (DG) and evaluation of the structural integrity of DG neurons by Nissl staining in cold-stressed mice. (**A**) GFAP expression in the DG. Scale bar = 50 μm. (**B**) Quantification of GFAP-positive cells in the DG. Differences between room temperature male (RTM) and cold exposure male (CEM), room temperature female (RTF) and cold exposure female (CEF), and CEM and CEF groups were evaluated by two-way analysis of variance. Results are expressed as the mean ± SD (*n* = 6) of four independent experiments. * *p* < 0.05, ***p < 0.001.

To determine the effects of cold exposure on glial cells in the hippocampus of mice, the expression of astrocytes and activated microglia was counted. IBA-1 and GFAP (Figure 2A) levels were increased in the CEM and CEF groups and IBA-1 expression was increased in the CEM group relative to the other two groups (Figure 2B,C), as determined by western blotting. Immunofluorescence analysis revealed that the number of CD11b- and IBA-1-positive cells in the dentate gyrus (DG) (Figure 3A,C) was increased in the DG region in the CEM and CEF groups, with a greater number of cells in the CEM group than in the CEF group (Figure 3B,D). The number of GFAP-positive astrocytes in the DG was higher in the CEM and CEF groups than in the other groups (Figure 4A), and was higher in the former than in the latter (*p* < 0.05; Figure 4B).

### 3.3. Cold Exposure Alters Hippocampus Homeostasis

**Figure 5:**
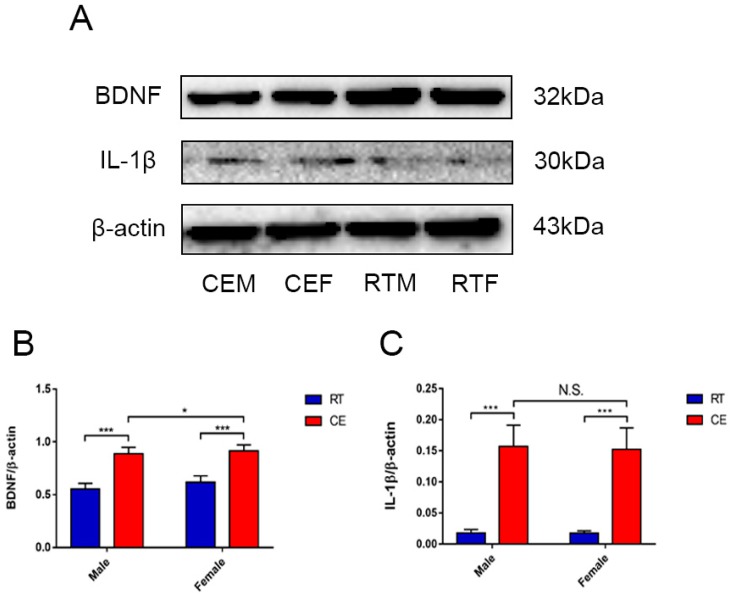
Neurotrophin and inflammatory cytokine expression in the hippocampus of cold-stressed mice. (**A**) Brain-derived neurotrophic factor (BDNF), interleukin (IL)-1β, and β-actin levels were evaluated by western blotting and quantified by densitometric analysis. (**B**,**C**) Quantification of expression levels based on BDNF/β-actin (**B**) and IL-1β/β-actin (**C**) ratios. Differences between room temperature male (RTM) and cold exposure male (CEM), room temperature female (RTF) and cold exposure female (CEF), and CEM and CEF groups were evaluated by two-way analysis of variance. Results are expressed as the mean ± SD (*n* = 6) of four independent experiments. N.S.: not significant; * *p* < 0.05, *** *p* < 0.001.

**Figure 6:**
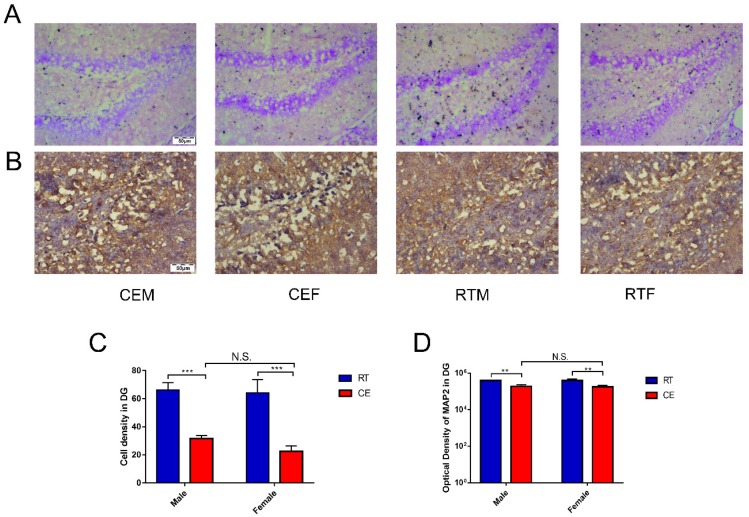
Expression of the neuronal dendrite marker microtubule-associated protein (MAP)2 in the dentate gyrus (DG) of cold-stressed mice. (**A**) MAP2 expression in the DG was detected by immunohistochemistry. Scale bar = 50 μm. (**B**) Representative Nissl staining of the DG region. Scale bar = 50 μm. (**C**) Quantification of the MAP2 expression level by densitometry. Differences between room temperature male (RTM) and cold exposure male (CEM), room temperature female (RTF) and cold exposure female (CEF), and CEM and CEF groups were evaluated by two-way analysis of variance. Results are expressed as the mean ± SD (*n* = 6) of four independent experiments. N.S.: not significant; ** *p* < 0.01. (**D**) Quantification of Nissl-positive cells in the DG. Differences between RTM and CEM, RTF and CEF, and CEM and CEF groups were evaluated by two-way analysis of variance. Results are expressed as the mean ± SD (*n* = 6) of four independent experiments. N.S.: not significant; *** *p* < 0.001.

**Figure 7:**
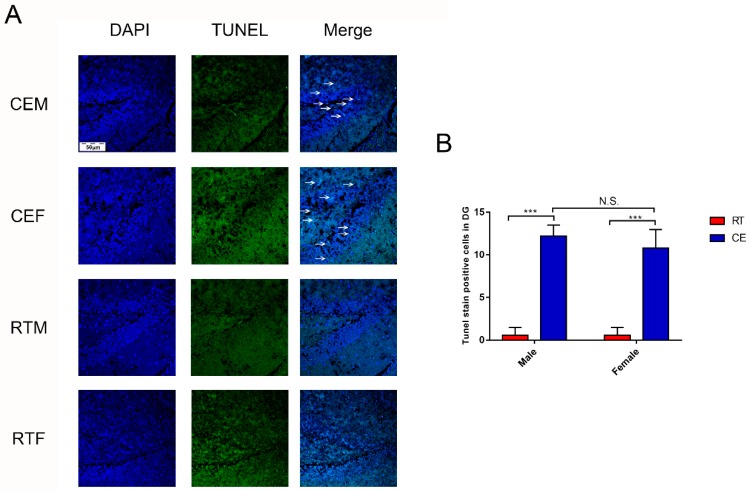
Apoptosis in the dentate gyrus (DG) of cold-stressed mice. (**A**) Transferase dUTP Nick End Labeling (TUNEL)-positive cells (green) counterstained with 4’,6-diamidino-2-phenylindole in the DG. Scale bar = 50 μm. (**B**) Quantification of TUNEL-positive cells. Differences between room temperature male (RTM) and cold exposure male (CEM), room temperature female (RTF) and cold exposure female (CEF), and CEM and CEF groups were evaluated by two-way analysis of variance. Results are expressed as the mean ± SD (*n* = 6) of four independent experiments. N.S.: not significant; *** *p* < 0.001.

Homeostasis in the hippocampus following cold exposure was evaluated by measuring the levels of the neurotrophin BDNF and pro-inflammatory cytokine IL-1β in the hippocampus by western blotting. BDNF was downregulated in the CEM and CEF groups relative to the RTM and REF groups (Figure 5A), and was decreased in the CEM group compared with the CEF group (Figure 5B). Conversely, IL-1β was upregulated in the CEM and CEF groups, with no significant difference observed between them (Figure 5C). Nissl staining revealed the presence of Nissl bodies in DG cells (Figure 6A); the CEM and CEF groups had fewer Nissl-positive cells than the RTM and REF groups, although they did not show a statistically significant difference with respect to each other (Figure 6C). Additionally, the CEM and CEF groups showed a reduced expression of MAP2 in the DG compared with the RT group by immunohistochemistry (Figure 6B). However, there was no difference in the levels between these two groups (Figure 6D). The TUNEL assay showed that the apoptosis of DG cells was increased in the CEM and CEF groups compared with the RTM and RTF groups (Figure 7A), although there was no difference between the CEM and CEF groups (Figure 7B).

### 3.4. Cold Exposure Increases HMGB1 and p65 Acetylation in the Hippocampus

**Figure 8:**
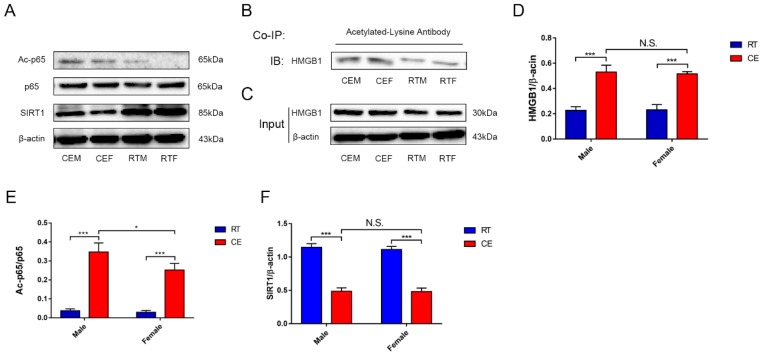
Expression of intracellular signaling pathway components in the hippocampus of cold-stressed mice. (**A**) Acetyl-p65 (Lys310), p65, silent mating type information regulation 2 homolog (SIRT)1, and β-actin levels were evaluated by western blotting. (**B**,**C**) High-mobility group box (HMGB)1 protein expression and acetylation in hippocampus tissue was evaluated by western blotting (**B**) and co-immunoprecipitation (co-IP) (**C**). (**D**–**F**) Quantification of expression levels based on HMGB1/β-actin (**D**), acetyl-p65/p65 (**E**), and SIRT1/β-actin (**F**) ratios. Differences between room temperature male (RTM) and cold exposure male (CEM), room temperature female (RTF) and cold exposure female (CEF), and CEM and CEF groups were evaluated by two-way analysis of variance. Results are expressed as the mean ± SD (*n* = 6) of four independent experiments. N.S.: not significant; * *p* < 0.05, ** *p* < 0.01, *** *p* < 0.001.

NF-κB activation was determined by measuring the level of acetyl-p65 and the expression of SIRT1 (Figure 8A). The results demonstrated that acetyl-p65 and SIRT1 were upregulated in the CEM and CEF groups compared with the RTM and RTF groups, with a higher level of p65 acetylation in the CEM group (Figure 8E,F). Additionally, HMGB1 expression was increased in both the CEM and CEF groups (Figure 8D), and co-IP revealed that HMGB1 acetylation was increased (Figure 8B).

### 3.5. Excess CORT Activates BV2 Cells

**Figure 9:**
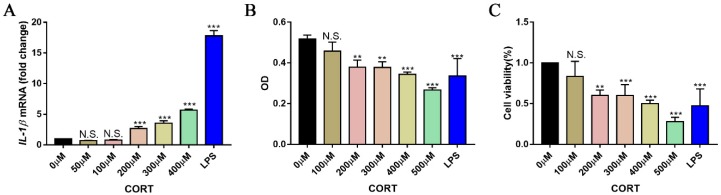
mRNA expression of inflammatory cytokines and effects of cortisol (CORT) on the viability of BV2 cells. (**A**) Interleukin (IL)-1β mRNA level in BV2 cells following treatment with indicated concentrations of CORT (0–400 μM) for 3 h was evaluated by quantitative real-time (qRT)-PCR; cells treated with lipopolysaccharides (LPS) (1 μg/mL) served as a positive control. Differences among control, CORT, and LPS groups were evaluated by one-way analysis of variance. Results are expressed as the mean ± SD (*n* = 3) of three independent experiments. N.S.: not significant; *** *p* < 0.001. (**B**,**C**) BV2 cells were treated with different concentrations of CORT (0–500 μM) for 3 h and viability was assessed with the cell counting kit (CCK)-8 assay, and cells treated with LPS served as a positive control. Differences among control, CORT, and LPS groups were evaluated by one-way analysis of variance. Results are expressed as the mean ± SD (*n* = 3) of three independent experiment. N.S.: not significant; ** *p* < 0.01; *** *p* < 0.001.

**Figure 10:**
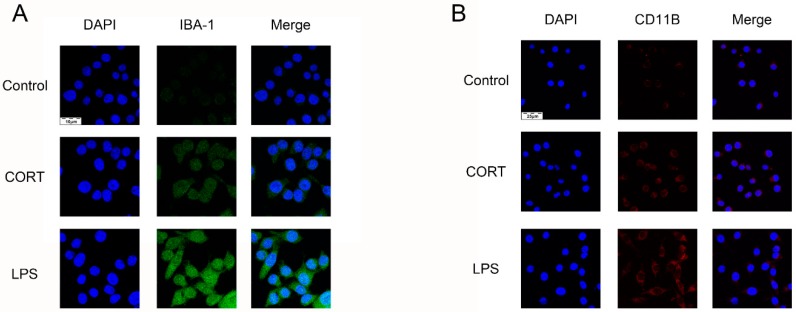
Detection of microglia activation following cortisol (CORT) treatment. (**A**,**B**) Expression of the activated microglia markers ionized calcium-binding adapter molecule (IBA)-1 and CD11b was detected by immunocytochemistry in cells with or without CORT treatment (200 μM) for 3 h. Cells treated with LPS (1 μg/mL) served as a positive control.

To determine whether BV2 cells are activated by excess CORT and establish the range of effective concentrations, we assessed the effect of CORT on BV2 cell viability and activation. Cells were treated with different concentrations of CORT (0–500 μM) for 3 h and the IL-1β level was measured by qRT-PCR; cell viability was evaluated with the CCK-8 assay, and glial cell activation was assessed by IBA-1 and CD11b immunofluorescence analysis. IL-1β expression was upregulated relative to the control in a concentration-dependent manner, with the highest expression observed at 200 μM (Figure 9A). The results of the CCK-8 assay showed that treatment with 200 μM CORT for 3 h decreased cell viability (Figure 9B,C). The number of IBA-1- and CD11b-positive cells was also increased by CORT treatment, as observed directly (Figure 10A,B).

### 3.6. Excess CORT Promotes HMGB1 Activation and Secretion in BV2 Cells

**Figure 11:**
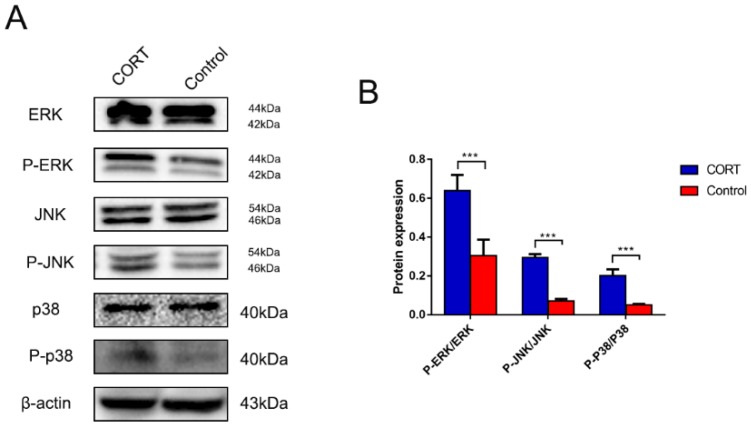
Expression of mitogen-activated protein kinase (MAPK) signaling pathway components in BV2 cells after cortisol (CORT) treatment (200 μM) for 3 h, as assessed by western blotting. (**A**) Extracellular signal-regulated kinase (ERK), phospho- (p-)ERK (Thr202/Tyr204), c-Jun N-terminal kinase (JNK), p-JNK (Thr183/Tyr185), p38, p-p38 (Thr180/Tyr182), and β-actin expression. (**B**) Quantification of expression levels based on p-ERK/ERK, p-JNK/JNK, and p-p38/p38 ratios. Differences between control and CORT groups were evaluated by one-way analysis of variance. Results are expressed as the mean ± SD (*n* = 3) of three independent experiments. *** *p* < 0.001.

**Figure 12:**
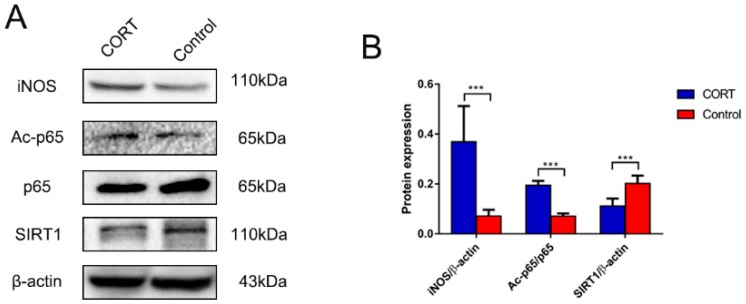
Acetylation and expression of nuclear factor (NF)-κB signaling pathway components in BV2 cells after cortisol (CORT) treatment (200 μM) for 3 h, as determined by western blotting. (**A**) iNOS, acetyl-p65 (Lys310), p65, SIRT1, and β-actin expression. (**B**) Quantification of expression levels based on inducible nitric oxide synthase (iNOS)/β-actin, acetyl-p65/p65, and silent mating type information regulation 2 homolog (SIRT)1/β-actin ratios. Differences between control and CORT groups were evaluated by one-way analysis of variance. Results are expressed as the mean ± SD (*n* = 3) of three independent experiments. *** *p* < 0.001.

**Figure 13:**
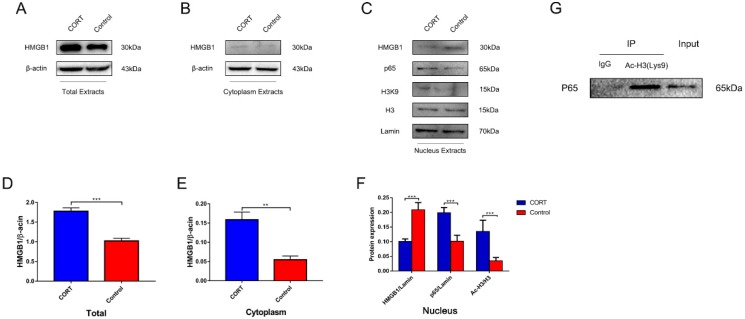
Expression of high-mobility group box (HMGB)1 in cortisol (CORT)-treated BV2 cells and interaction between H3K9 and p65. (**A**–**C**) Expression levels of HMGB1 in total cell (**A**) and cytoplasm (**B**) and HMGB1, p65, H3K9, histone H3, and lamin expression (**C**) in nuclear extracts after CORT (200 μM) treatment for 3 h were evaluated by western blotting. Quantification of HMGB1, acetyl-p65, p65, H3K9, histone H3, and β-actin expression levels based on HMGB1/β-actin (**D**), HMGB1/β-actin (**E**), HMGB1/β-actin, p65/lamin, and H3K9/histone H3 (**F**) ratios. Differences between control and CORT groups were evaluated by one-way analysis of variance. Results are expressed as the mean ± SD (*n* = 3) of three independent experiments. ** *p* < 0.01, *** *p* < 0.001. (**G**) Co-immunoprecipitation (Co-IP) of acetyl-histone H3 (Lys9) and p65 in BV2 cells with or without CORT treatment. Right to left, whole cell extracts (lane 1), immunoprecipitate obtained with anti-acetyl-histone H3 (Lys9) antibody (lane 2), and control IgG (lane 3) were probed with the anti-HMGB1 antibody.

To clarify the mechanism by which excess CORT activates BV2 cells, the levels of mitogen-activated protein kinase (MAPK) family proteins (Figure 11A) and nuclear factor (NF)-κB (Figure 12A) signaling pathway components were evaluated by western blotting and co-IP. The phosphorylation of MAPK family member ERK (Thr202/Tyr204), JNK (Thr183/Tyr185), and p38 (Thr180/Tyr182) was increased after 200 μM CORT treatment, as determined by western blotting (Figure 11B). Moreover, iNOS expression and p65 Lys310 acetylation were increased and SIRT1 expression was decreased in whole-cell extracts (Figure 12B), whereas p65 expression and H3 Lys9 acetylation were increased in the nucleus in the presence of excess CORT. A co-IP analysis revealed that CORT promoted the interaction between acetyl-H3 (Lys9) and p65 (Figure 13G).

**Figure 14:**
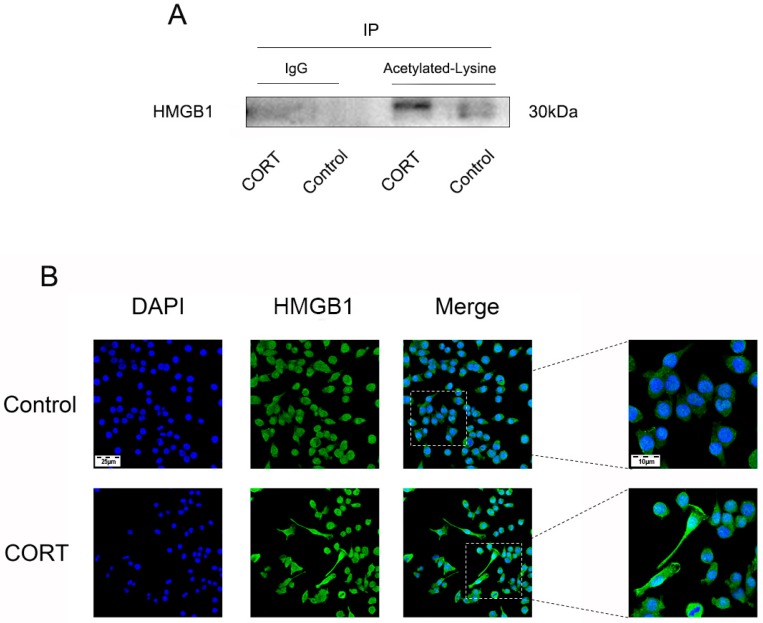
High-mobility group box (HMGB)1 acetylation level and protein localization in cortisol (CORT)-treated BV2 cells. (**A**) Co-immunoprecipitation (Co-IP) of acetyl-Lys with HMGB1 from BV2 cells with or without CORT treatment (200 μM) for 3 h. Right to left, immunoprecipitates obtained with acetyl-histone H3 (Lys9) antibody (lanes 1 and 2) or control IgG (lanes 3 and 4) were probed with an anti-HMGB1 antibody. (**B**) HMGB1 protein localization in BV2 cells, as detected by immunocytochemistry.

We next examined the mechanism by which HMGB1 is released (Figure 13A–C) and found that HMGB1 expression was increased in whole-cell (Figure 13D) and cytoplasmic (Figure 13E) extracts, but was reduced in nuclear extracts (Figure 13F) following treatment with 200 μM CORT. The results of the co-IP assay indicated that HMGB1 acetylation was increased by CORT treatment (Figure 14A) and immunofluorescence analysis revealed that HMGB1 was translocated from the nucleus to the cytoplasm under this condition (Figure 14B).

## 4. Discussion

This is the first systematic demonstration of a potential link between cold stress and neuroinflammation in the brain. The in vivo results showed that chronic cold exposure induced stress in mice, resulting in microglia activation and neuroinflammation, which reduced the integrity of neuronal structures in the hippocampus. In our previous study, we demonstrated that the CORT level was significantly increased both in serum and hippocampus-mediated neuronal apoptosis via destruction of the function of mitochondria [21]. Additionally, the structure was destroyed in CA1 and CA3 regions of the hippocampus due to neuroinflammation significantly inducing neuronal loss and influencing the behavior following cold exposure [12]. The results all indicated that the negative effect on mice was closely related to the impact on the DG region of the hippocampus following cold exposure. Based on these observations, we speculated that cold stress impairs the DG region of the hippocampal function via microglia-mediated HMGB1 acetylation and secretion induced by hyperactivation of the HPA axis causes the disruption of homeostasis due to abnormal glucocorticoid secretion, resulting in neuroinflammation and neuronal apoptosis. To test this hypothesis, we investigated the effect of CORT exposure on microglia activation and HMGB1 release using BV2 cells. We found that CORT activated MAPK signaling, which promoted the acetylation of NF-κB via increased HMGB1 acetylation and nuclear translocation.

The chronic cold stress model was established based on our previous report [19]. First, the level in the serum and hippocampus was measured to confirm our hypothesis. The results demonstrated that the level was significantly increased both in the serum and hippocampus. Furthermore, we confirmed that cold stress-activated glial cells in the DG of the hippocampus, as evidenced by the upregulation of IBA-1 and GFAP proteins in the CEM and CEF groups. This was further substantiated by the observation that CD11b, IBA-1, and GFAP immunoreactivity was increased in the DG, which was accompanied by an increase in the level of the pro-inflammatory cytokine IL-1β and downregulation of BDNF. Interestingly, glial cell activation was higher in the CEM than in the CEF group. Glial cells regulate brain homeostasis [22] and their activation indicates the dysregulation of this process. Since the DG is associated with emotion and memory functions [23], this could lead to higher levels of anxiety. In our previous study, we showed that cold exposure increased serum and hippocampus glucocorticoid levels and activated microglia in the CA1 and CA3 regions of the hippocampus, as well as in the DG. This was accompanied by increased anxiety-like behavior [12], which confirms the findings of our previous study [19] and other reports [24,25].

The dual role of microglia has been widely investigated. In addition to maintaining homeostasis, activated microglia release damage-associated molecular patterns (DAMPs) and proinflammatory factors that alter neuronal signaling [26]. In the present study, our immunohistochemical analysis revealed that MAP2 expression was reduced, whereas the number of TUNEL-positive cells in the hippocampus, reflecting DNA fragmentation in apoptotic cells [27], was increased in the CEM and CEF groups. The results of the TUNEL assay were supported by Nissl staining, which uses an aniline stain to label extranuclear RNA granules in neuronal cell bodies [28]. Our results show that there were fewer Nissl-positive cells in the hippocampus of mice exposed to cold stress than in controls, indicating significant neuronal loss as a result of cold exposure. Other studies have demonstrated sex differences in response to stress; for instance, estradiol and oxytocin play important roles in protection from stress and stress avoidance. In our previous study, we showed that the levels of neurotransmitter metabolites in the hippocampus differed between male and female mice, which could also contribute to the observed difference in the response to stress between sexes.

Activated microglia secrete DAMPs, which induce inflammation through the innate immune response. HMGB1 is a type of DAMP that is released upon acetylation and activates NF-κB signaling [29]. HMGB1 expression was elevated in the hippocampus of mice after cold exposure and the co-IP results indicated that HMGB1 acetylation was increased. Meanwhile, the NAD^+^-dependent histone deacetylase SIRT1 [30] was downregulated, suggesting that acetylation was enhanced. This was supported by the increase in the level of acetyl-p65, the active form of NF-κB, which promotes glial cell activation and neuroinflammation. The increased secretion of acetylated HMGB1 provided further evidence that glial cells were activated and brain homeostasis was disrupted [31].

To evaluate the mechanism underlying microglia activation by cold stress, BV2 cells were treated with different concentrations of CORT to establish an in vitro stress model corresponding to the time course of cold stress exposure in mice. IL-1β mRNA expression was upregulated and cell viability was reduced in a time-dependent manner by treatment with 200 μM CORT. Activated microglia are the main source of IL-1β in the brain [32]. Therefore, our results indicated that 200 μM CORT activated microglia. This was confirmed by the increased number of CD11b- and IBA-1-positive cells after 200 μM CORT treatment.

We next examined intracellular signaling in BV2 cells induced by treatment with 200 μM CORT. MAPK pathway activation was investigated by evaluating the phosphorylation level of pathway components by western blotting, and we found that ERK, JNK, and p38 phosphorylation was increased. Various stressors activate MAPK signaling, and glucocorticoids are known to interact with MAPKs [33,34]. The p65 acetylation and nuclear translocation, reflecting NF-κB activation, were increased by CORT treatment, whereas SIRT1 expression was decreased. MAPKs act upstream of NF-κB signaling and their activation leads to NF-κB-induced iNOS secretion and inflammation [35].

HMGB1 is released by activated microglia. In our study, western blot analysis revealed that HMGB1 expression was increased in total cell and cytoplasmic extracts, but reduced in nuclear extracts. Moreover, histone H3 (Lys9) acetylation and interaction with p65 were increased by CORT treatment, which could promote HMGB1 exocytosis, a process that was shown to involve microglia [36]. These data and those of the co-IP assay confirmed that HMGB1 expression and acetylation were increased following CORT exposure, suggesting that HMGB1 secretion was elevated. Therefore, activated NF-κB interacted with acetylated histone H3 (Lys9), inducing the activation of microglia and promoting HMGB1 acetylation and release, as previously reported [37].

**Figure 15:**
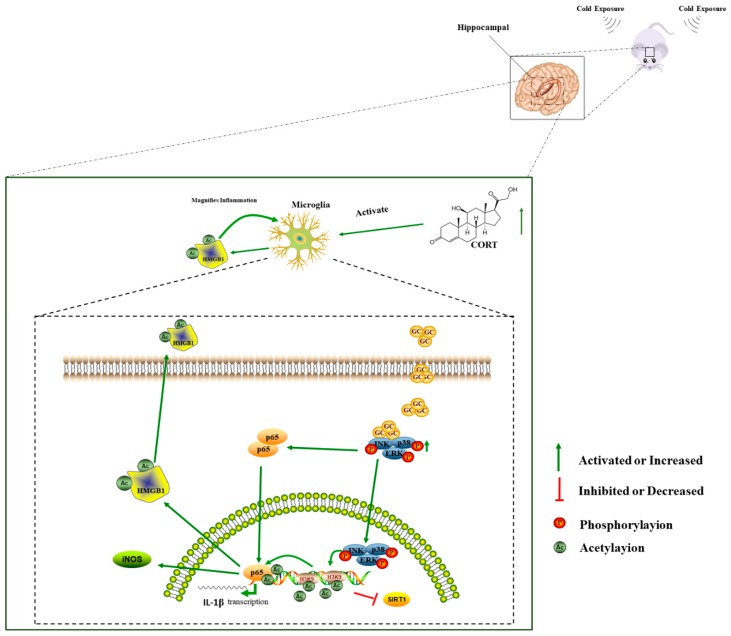
Proposed model for the mechanisms of microglia activation induced by cold stress in the hippocampus.

Our in vitro findings demonstrated that repeated CORT exposure has the same effects as chronic cold exposure in mice; that is, glial cells are activated, leading to neuroinflammation and neuronal apoptosis (Figure 15). Moreover, acetylation played a key role in hippocampal responses to cold stress and strategies that inhibit this process may reduce the negative effects of cold stress in the brain, a possibility that we intend to investigate in future studies. Finally, our results provide evidence for sex differences in the response to cold stress in mice, which may determine the selection of animal models in future stress-related studies.

## 5. Conclusions

Chronic cold exposure caused stress-induced HPA axis activation and CORT release, which induced glial cell activation and resulted in increased neuroinflammation and neuronal apoptosis and structural damage to the DG of the hippocampus. Increased acetylation caused by high levels of CORT activated MAPK signaling, NF-κB and microglia activation, and HMGB1 acetylation and release. We also observed sex differences in the response to cold stress, with a greater degree of DG glial cell activation and neuroinflammation in male mice. These findings clarified the mechanisms underlying the cold stress response and provided a basis for investigating new strategies to combat the effects of hypothermia.

## Figures and Tables

**Table 1:** Primer sequences for PCR amplification of IL-1β and β-actin.

Item	Primer	Lengh (bp)
IL-1β (Forward)	5’-GCTGCTTCCAAACCTTTGAC-3’	121
IL-1β (Reverse)	5’-AGCTTCTCCACAGCCACAAT-3’	
β-actin (Forward)	5’-GTCAGGTCATCACTATCGGCAAT-3’	147
β-actin (Reverse)	5’-AGAGGTCTTTACGGATGTCAACGT-3’	
